# Modulation of Synaptic Vesicle Exocytosis in Muscle-Dependent Long-Term Depression at the Amphibian Neuromuscular Junction

**DOI:** 10.1371/journal.pone.0087174

**Published:** 2014-01-28

**Authors:** Sarah J. Etherington, Victoria P. A. Johnstone, Alan W. Everett

**Affiliations:** 1 School of Biomedical, Biomolecular and Chemical Sciences, The University of Western Australia, Perth, Western Australia, Australia; 2 School of Veterinary and Life Sciences, Murdoch University, Perth, Western Australia, Australia; 3 Department of Physiology, Monash University, Clayton, Victoria, Australia; University of Sydney, Australia

## Abstract

We have labeled recycling synaptic vesicles at the somatic *Bufo marinus* neuromuscular junction with the styryl dye FM2-10 and provide direct evidence for refractoriness of exocytosis associated with a muscle activity-dependent form of long-term depression (LTD) at this synapse. FM2-10 dye unloading experiments demonstrated that the rate of vesicle exocytosis from the release ready pool (RRP) of vesicles was more than halved in the LTD (induced by 20 min of low frequency stimulation). Recovery from LTD, observed as a partial recovery of nerve-evoked muscle twitch amplitude, was accompanied by partial recovery of the refractoriness of RRP exocytosis. Unexpectedly, paired pulse plasticity, another routinely used indicator of presynaptic forms of synaptic plasticity, was unchanged in the LTD. We conclude that the LTD induces refractoriness of the neuromuscular vesicle release machinery downstream of presynaptic calcium entry.

## Introduction

Retrograde signaling is central to the development, survival and activity-dependent modulation of synaptic connections [1], [2]. Signaling from post- to pre-synaptic cells is required for several Hebbian forms of synaptic plasticity, believed to underlie learning and memory [3]. More recently, retrograde signaling has received renewed attention for its role in homeostatic plasticity. In this case, postsynaptic detection of perturbations to transmission initiates precise negative-feedback loops that restore transmission to baseline levels [4].

A number of postsynaptic signaling pathways involved in activity-dependent modulation of vertebrate neuromuscular transmission are particularly well described. At the lizard NMJ, muscle-derived endocannabinoids (eCB) generated by sustained low-frequency synaptic activity or activation of M3 muscarinic receptors induce depression of transmitter release. Similarly, ATP, a known inhibitor of presynaptic secretion [5], [6], is released from skeletal muscles in an activity-dependent manner [7]. Skeletal muscle activity also produces nitric oxide (NO) mediated depression at the amphibian neuromuscular junction in response to postsynaptic metabotropic glutamate receptor (mGluR) activation [8] or skeletal muscle action potential firing [9]. Retrograde signaling pathways involving a complex interplay between cannabinoid, prostanoid and NO signaling [10], [11], as well as distinct mechanisms activated by muscle-derived adenosine [12], have also been implicated in the activity-dependent enhancement of neuromuscular transmission.

By comparison, the presynaptic targets of retrograde neuromuscular signaling pathways are poorly described. Commonly, a presynaptic locus for depression of evoked release has been inferred from electrophysiological recordings showing reduced frequency but not amplitude of spontaneous synaptic events [11], [13], [14]. More precise investigations of presynaptic signaling targets have generally used pharmacological agents to mimic endogenous retrograde signaling pathways. For example, Newman *et al*. [15] used calcium imaging to document a reduction in the single AP calcium transient in response to exogenous cannabinoids, and suggest this mechanism may contribute to eCB-mediated depression at the NMJ (see also [16]). Contrastingly, NO donors that depress EPP amplitude at the amphibian NMJ do not lead to a decrease in resting or activity-dependent calcium levels; indeed the amplitude of presynaptic calcium transients increased in presence of a NO donor during high frequency stimulation [17]. These studies suggest that there are likely to be multiple presynaptic targets for retrograde modulation of transmitter release at the NMJ; shedding light on the presynaptic mechanisms underlying one such retrograde signaling pathway was the aim of the present work.

We have previously characterized a presynaptic form of long-term depression (LTD) elicited by sustained low-frequency stimulation (1 Hz for 20 min [13]). The LTD, which lasts > 2 hours, is NO-dependent, with postsynaptic AP firing both necessary and sufficient to induce reduction in transmitter release. This unqualified dependence on postsynaptic AP firing distinguishes the LTD from most presynaptic forms of plasticity expressed at the vertebrate NMJ and suggests that it may play a novel role in modulating network function [9].

Here, we combine electrophysiological recordings with optical labeling to directly monitor vesicle recycling during this muscle AP-dependent form of neuromuscular LTD. Styryl dye labeling revealed refractory exocytosis of readily releasable vesicles during LTD, however short-term synaptic plasticity was unchanged. We conclude that this form of LTD acts subsequent to the entry of calcium to the terminal. Implications for the reliable control of motor function are discussed.

## Materials and Methods

### Ethics Statement

All experiments were performed in accordance with the Australian Code of Practice for the Care and Use of Animals for Scientific Purposes (7th edition, 2004). The work was approved by the Animal Ethics Committee at The University of Western Australia (Permit 4/100/325).

### Animals And Dissection

Iliofibularis muscles with nerve supplies intact were isolated from young cane toads following euthanasia by double pithing, as described previously [13]. Unless otherwise indicated, preparations were maintained at room temperature (21–24°C), in a modified aerated amphibian Ringer solution containing (mM) NaCl (114), KCl (2), glucose (5), NaHCO_3_ (1.8), 3-(N-Morpholino)propanesulfonic acid (10) and CaCl_2_ (1.5), pH adjusted to 7.4 with NaOH.

### Induction Of Long-Term Depression

LTD was induced using repetitive sciatic nerve stimulation with a platinum-iridium suction electrode (1 Hz stimulation for 20 min with 1 ms square pulses at optimum voltage), as described previously [13]. Unless otherwise stated, LTD was induced in one iliofibularis muscle from an animal and the other iliofibularis from the same animal was used as a control.

### Electrophysiology

Synaptic and action potentials were recorded from iliofibularis muscles with sharp borosilicate glass electrodes (R = 7–20 MΩ) containing 3 M KCl, according to published methods [9], [13]. End-plate potentials (EPPs) were recorded in the presence of *d*-tubocurarine chloride (0.6–1.6 µM, Sigma) to minimize muscle contraction. APs were recorded in the presence of the myosin ATPase inhibitor *N*-benzyl-*p*-toluene sulfonamide (BTS, 50 µM, Sigma Rare Chemicals). All recordings were performed within 1 hr of induction of LTD.

### Fm2-10 Staining And Destaining

Terminal labeling was achieved by sciatic nerve stimulation in the presence of FM2-10 (70- 200 µM, Molecular Probes, Oregon, USA) using a platinum iridium suction electrode.

Vesicle pools were selectively labeled using protocols validated at the amphibian neuromuscular junction [18], [19]. The protocols utilize immediate post-stimulation dye removal to label the RRP, as the relatively hydrophilic FM2-10 dye partitions easily from lipid bilayers. In contrast, leaving preparations in dye for several minutes following stimulation gives time for internalization of the dye by a larger recycling pool, equivalent to that labeled by more lipophilic FM dyes [19]. The total recycling pool (TP) was therefore labeled by prolonged stimulation in the presence of the dye (30 Hz for 2.5 min), after which the preparation was left in the dye for an additional 20 min (delayed wash protocol [19]). The intensity of fluorescent labeling was unchanged by an additional 60 sec of nerve stimulation in the presence of the dye (data not shown). The RRP of vesicles was labeled to saturation by stimulating the sciatic nerve at 30 Hz for 15 sec in the presence of the dye and then immediately rinsing the preparation with dye-free Ringer solution (immediate wash protocol [19]). After dye loading, all preparations were washed in Ringer solution at 4°C for at least one hour before imaging.

For destaining experiments, muscles were pinned out in Sylgard-lined dishes in a dye-free solution of *d*-tubocurarine chloride (7–10 µM, Sigma) to limit muscle contraction. The *d*-tubocurarine chloride was only applied *after* the LFS protocol used to induce LTD; we have shown previously that post-stimulation application of the antagonist does not interfere with expression of LTD, although presence of the antagonist during LFS blocks depression [9], [13]. A labeled nerve terminal was positioned in dye-free medium within the field of view, imaged, and then sciatic nerve stimulation (30 Hz for 10 sec, 1 ms square pulses) was delivered via a suction electrode to destain terminals. Each preparation was exposed to two 10 sec destaining periods, separated by interval of at least 3 min.

### Capture And Analysis Of Fluorescent Images

FM2-10 labeled terminals in living muscles were visualized with a 40× water dipping objective on a Nikon E600FN upright microscope. Excitation light was delivered by a 100 W mercury lamp (Lab Supply) through an Olympus B-2E/C filter block (excitation filter 465–495 nm, dichroic mirror 505 nm and emission filter 515–555 nm). Fluorescent 8-bit images were captured with a Spot-RT CCD camera (Diagnostic Instruments) mounted on the microscope and controlled by SPOT RT software (version 4.0.2, Diagnostic Instruments). A Uniblitz external shutter system (Vincent Associates) controlled exposure time.

For each terminal a z-series of images at 0.5 µM intervals was captured, covering the full range of focal depths of the terminal branches. The z-series stack was collapsed into a single montage image using Auto-Montage Pro deconvolution software (fixed method optimized for precision, version 5.01.005, Synoptics). All image analysis was performed on the montage images using Scion Image (version Beta 4.0.2, Scion Corporation).

Styryl dye loading produces punctate labeling of amphibian motor nerve terminals [20]. The average fluorescent intensity of a 25 square pixel box centered on the most intense pixel in a punctum, multiplied by the selection size, was used to estimate total punctum intensity. All intensities were corrected for background fluorescence (estimated from the median intensity of terminal-free muscle fiber labeling). Punctum intensities were also corrected for variation in the gain and exposure settings of the CCD camera (adjusted for each terminal within a linear range). The intensity of at least 80 fluorescent puncta, taken from a minimum of 4 different nerve terminals, was used to calculate the average intensity of fluorescent labeling for each nerve-muscle preparation. In destaining experiments, the intensity of the same puncta was measured in images taken before destaining and after each destaining stimulus. Preparations were left to relax for 2 min between the end of the tetanus and imaging.

### Statistics

Results are expressed as mean ± SEM and P values less than or equal to 0.05 were considered statistically significant.

Statistical analysis of paired pulse facilitation and FM2-10 labeling experiments was performed using the Mixed procedure in SAS software (version 9.1, SAS Institute Inc., Cary, NC). Differences between least squares means were calculated for each combination of factors and two-tailed tests of least squares mean differences were performed where appropriate. The fixed and random effects investigated for each block of experiments were as follows;

*Paired pulse facilitation*: The effects of inter-stimulus interval (20, 40 and 60 ms) and condition (control and low-frequency stimulated) on paired pulse ratio were modeled. Muscle and animal were treated as random effects.

*FM2-10 labeling*: For comparisons of RRP and TP size, the intensity of dye labeling was modeled as a function of condition (control vs. low-frequency stimulated) and pool labeled (RRP vs. TP). In a separate set of experiments, RRP size was modeled as a function of condition (control vs. low-frequency stimulated) and duration of the FM2-10 loading tetanus (7.5 vs. 15 seconds).

## Results

### Paired Pulse Plasticity Is Normal In Presynaptic Neuromuscular Ltd

**Figure 1:**
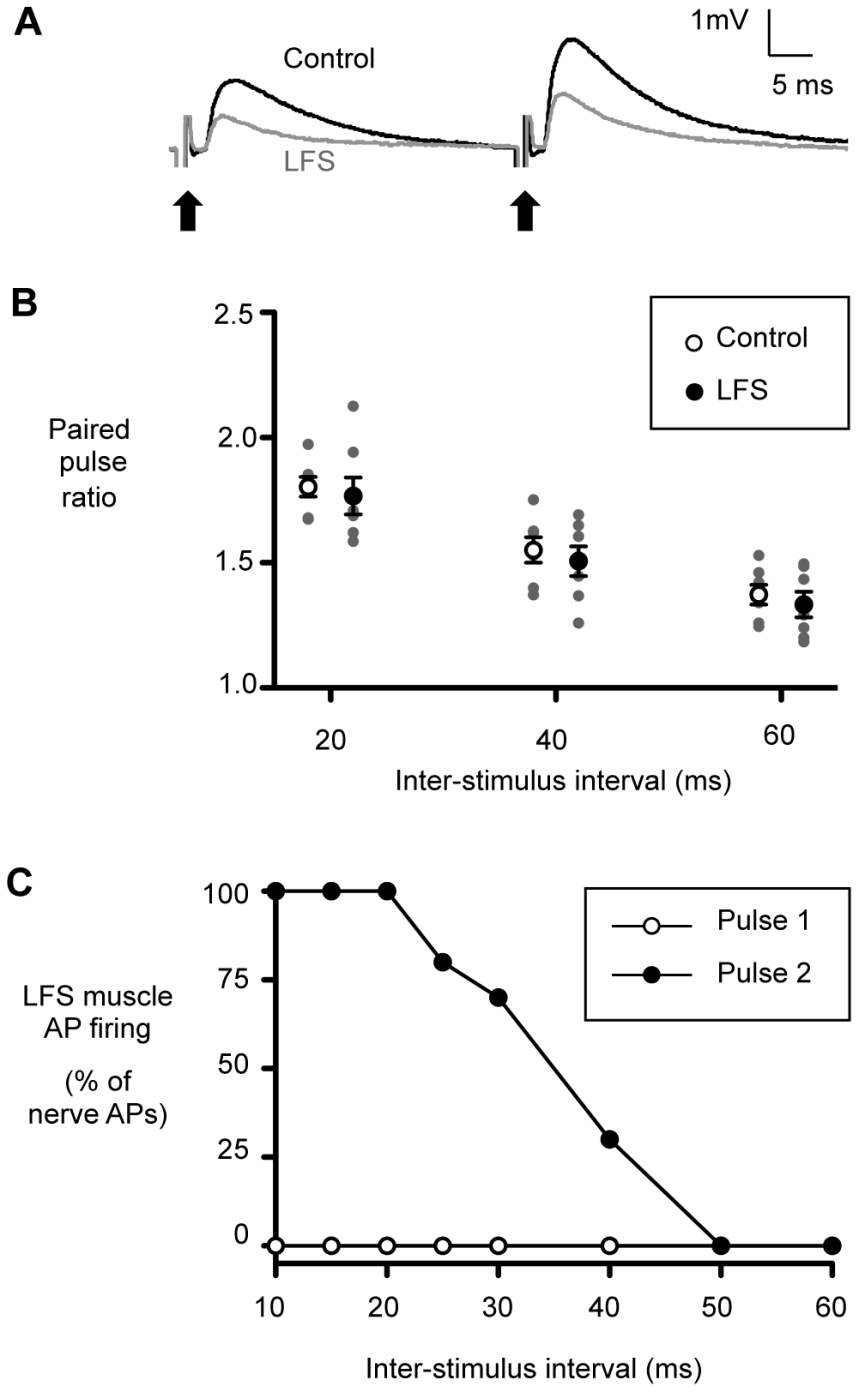
Facilitation is normal in LTD and restores muscle action potential firing in depressed preparations. *A*, representative traces of EPPs recorded from a control (black trace) and a low-frequency stimulated (grey trace) muscle in response to a pair of stimuli (arrows, 40 ms ISI), showing facilitation of the responses to the second stimulus in each pair. *B*, there was no difference in the level of facilitation, expressed as a paired pulse ratio, between control and LFS terminals at any of three ISIs tested (*n* = 7 pairs of muscles, F-test for main effect of site from mixed model analysis, *P* = 0.323). *C*, average level of muscle action potential firing following LFS evoked in a representative muscle fiber by pairs of nerve stimuli with varying ISIs. Muscle action potentials were never observed in response to the first nerve stimulation in a pair (open circles), consistent with the induction of LFS-dependent synaptic depression. Despite the intense depression, strong PPF at short ISIs (≤20 ms) was sufficient to completely restore the reliability of neuromuscular transmission.

The level of paired pulse facilitation (PPF) is inversely proportional to the initial release probability [21], thus an increase in PPF during LTD is commonly used as evidence for a presynaptic change in transmitter release [22]. We measured the level of PPF at three different inter-stimulus intervals (ISIs, 20, 40 and 60 ms) in control preparations and in preparations where LTD had been induced by 20 minutes of low-frequency nerve stimulation (LFS, see [13]). Median EPP amplitude in response to the first action potential in a pair was 47% lower in LFS preparations than in controls, consistent with the induction of LTD by LFS (see representative traces in Fig. 1*A* and also [13]). Unexpectedly, we found no difference in the paired pulse ratio between control and LFS (depressed) preparations (Fig. 1*A, B*). Paired-pulse facilitation was observed at all ISIs tested in control and LFS preparations, and was sufficient to restore muscle AP firing at short ISIs in profoundly depressed terminals (Fig. 1*C*). However, we did not observe the increased PPF commonly associated with presynaptic forms of LTD.

### While Vesicle Pool Size Is Unchanged In Ltd, Rrp Labeling Is Refractory

The observation that PPF is normal in LTD was somewhat unexpected, given our earlier conclusion that this form of depression is expressed presynaptically (from recordings of spontaneous synaptic potentials, [13]). To clarify the locus of expression of LTD, we used fluorescent styryl dye labeling, which is altered in a range of presynaptic forms of plasticity [23], [24], to directly monitor vesicle recycling.

**Figure 2:**
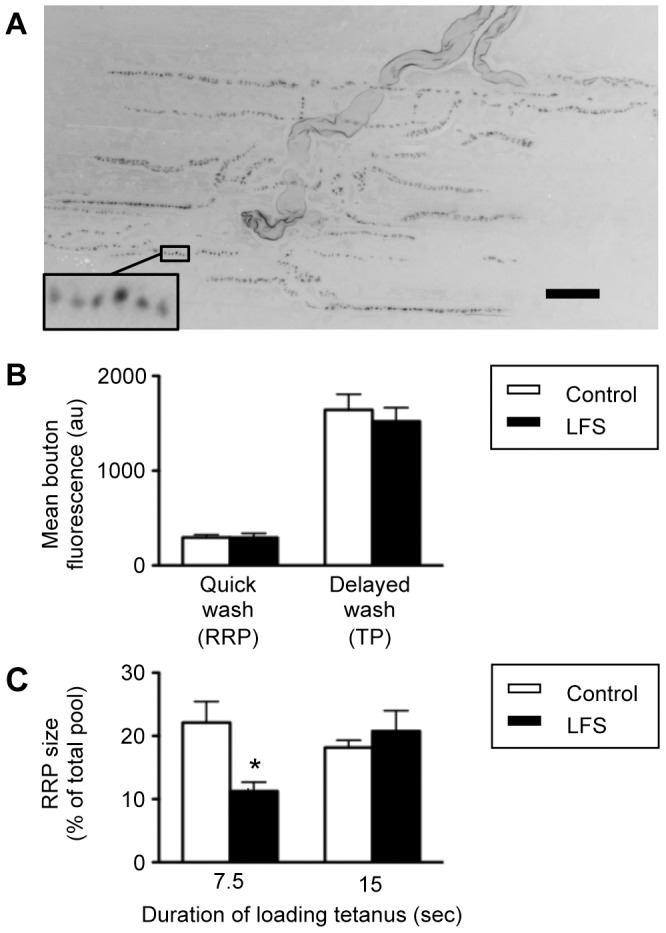
Loading of readily releasable pool vesicles with FM2-10 is refractory in LFS preparations. *A*, end-plate zone of amphibian skeletal muscle fibers showing nerve terminals where the RRP has been labeled to saturation with FM2-10. All nerve terminals within the focal range exhibit punctate staining associated with vesicle release sites. Inset is an enlarged section of a terminal branch, showing the fluorescent puncta analyzed to quantify FM2-10 labeling. Scale bar 20 microns. *B*, there was no difference in the intensity of FM2-10 fluorescence between control (open bars, *n* = 9 muscles) and low-frequency stimulated (LFS, filled bars, *n* = 11 muscles) terminals when synaptic vesicle pools were labeled to saturation (F-test of main effect of condition from mixed model analysis, *P* = 0.621). The quick wash protocol selectively labels the readily releasable pool (RRP), while the delayed wash labels the total recycling pool (TP). *C*, There was no increase in FM2-10 fluorescence associated with labeling of the RRP in control terminals (open bars) between 7.5 (*n* = 8 muscles) and 15 seconds (*n* = 9 muscles) of tetanic stimulation. In contrast, labeling of the RRP in low-frequency stimulated terminals was significantly lower after 7.5 seconds of tetanic stimulation (*n* = 6 muscles) than after a 15 second loading tetanus (*n* = 11 muscles, *, difference of least squares means *P*<0.05).

We followed well-established protocols [19] to selectively label either the readily releasable pool of vesicles (RRP) or the total recycling pool (TP) with the styryl dye FM2-10 (Fig. 2*A*). There was no difference in the overall fluorescent intensity of either RRP or TP labeling between control and LFS (depressed) neuromuscular preparations (Fig. 2*B*). In both cases, the RRP constituted approximately 20% of the total recycling pool, consistent with previous reports at this synapse [19]. Thus LTD does not affect the total number of vesicles available for recycling or the proportion of those vesicles that are available for immediate release from the nerve terminal.

The RRP is thought to maintain transmission at the amphibian neuromuscular junction at low frequencies [18]. We therefore focused our investigations on the RRP, varying the duration of the loading tetanus to investigate the time required for saturation of this pool with the styryl dye (Fig. 2*C*). Labeling in control nerve terminals did not increase between 7.5 and 15 seconds of tetanic stimulation in the presence of the dye (open bars, Fig. 2*C*), consistent with assertions that neuromuscular transmission during the first 15 seconds of a tetanus is mediated entirely by the small RRP of vesicles, without recruitment of vesicles from the reserve pool [19]. By contrast, RRP labeling in LFS preparations was approximately 50% lower following a 7.5 s loading tetanus, compared to the level of loading observed following a 15 s tetanus (filled bars, Fig. 2*C*). A similar trend was observed with an even shorter loading time of 3 seconds (*n* = 4, data not shown), although low intensity labeling made accurate quantification of fluorescence difficult. We conclude that vesicle recycling is refractory in LTD, increasing the time required to label the RRP to saturation.

### Ltd Is Associated With A Reversible Decrease In Rrp Exocytosis

From our previous work showing an LTD-associated decrease in the frequency of spontaneous synaptic potentials [13], we had hypothesized that the LTD involves a refractoriness of the exocytotic pathway. To examine this hypothesis experimentally the RRP was loaded to saturation in control and LFS preparations and we then quantified FM2-10 unloading during stimulation in dye-free medium (a measure of the level of exocytosis [25]).

**Figure 3:**
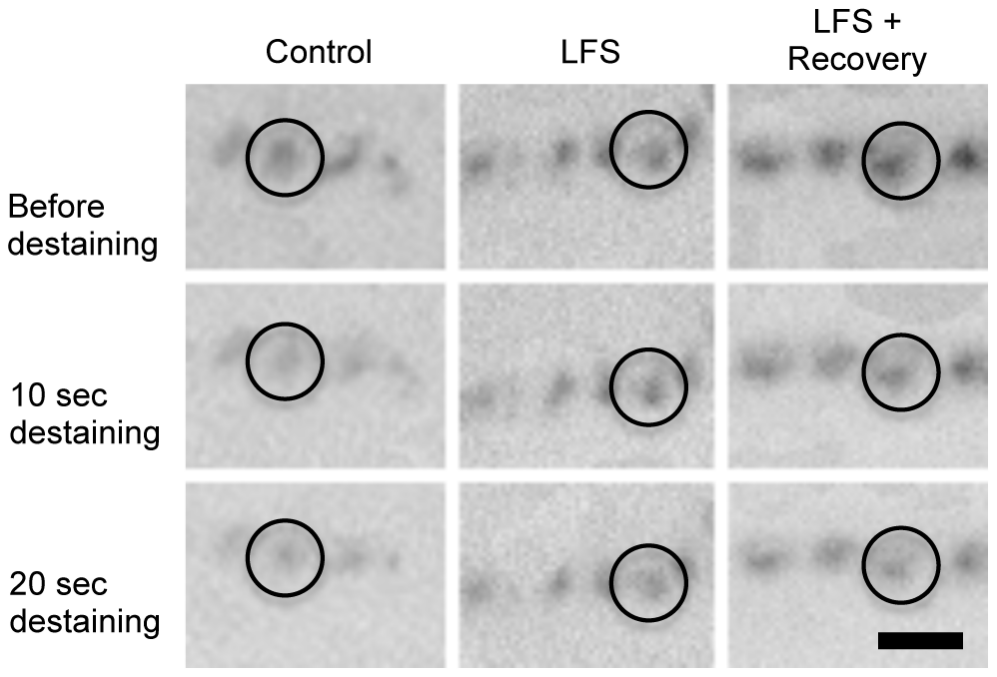
LFS is observed as a reversible reduction in vesicle exocytosis from the readily releasable pool. Puncta reflecting FM2-10 labeled readily releasable pool vesicles in motor nerve terminals before destaining (top row), after a single destaining stimulus (30 Hz stimulation for 10 sec in dye-free medium, middle row) and after two consecutive destaining stimuli (bottom row). Destaining was compared between control preparations, low-frequency stimulated (LFS) preparations, and LFS preparations that were left for two hours to recover before destaining (LFS + recovery). An individual punctum in each image is circled to assist visual comparison of destaining in the three conditions. Scale bar 5 microns.

Loaded terminals were exposed to two consecutive destaining stimuli (each involving 30 Hz stimulation in dye free medium for 10 seconds). Terminals were imaged before and immediately after each of the destaining stimuli and the same puncta were identified in each of the images (see representative examples in Fig. 3, relationship between puncta and nerve terminal branches illustrated in Fig. 2*A*), to quantify the amount of exocytosis at each time point. On average, the intensity of control puncta following the first destaining stimulus was 76±3% of the initial level, falling to 57±2% following the second destaining stimulus (*n* = 274 puncta sampled from 12 preparations). Strikingly, the level of FM2-10 destaining from LFS puncta (*n* = 161 puncta from 6 preparations) was less than half of that seen in controls, reaching only 91±1% and 81±2% of the initial level after the first and second destaining stimuli respectively (two-way ANOVA showed a significant condition x destaining time interaction, p<0.0001, Bonferroni post-tests revealed significantly less destaining from LFS preparations with both 10 s and 20 s of destaining, p<0.0001 in both cases).

Further FM2-10 destaining experiments revealed significant restoration of synaptic vesicle exocytosis when preparations were left to recover for 2 hours following a period of LFS. The average labeling in recovered preparations fell to 84±3% and then 69±3% of the initial level after the two destaining stimuli (*n* = 155 puncta from 6 preparations). This FM2-10 unloading from recovered LFS preparations was significantly greater than seen in LFS preparations that were unloaded immediately following the LFS routine (two-way ANOVA showed a significant condition x destaining time interaction, p<0.0001, Bonferroni post-tests revealed significantly less destaining from LFS preparations with both 10 s and 20 s of destaining, p<0.05 and p<0.001 respectively). We observed significant recovery of the nerve-evoked muscle twitch over the same period (from ∼20% to 42±7% of baseline, *n* = 6 muscles). We conclude that the depression of synaptic vesicle exocytosis induced by LFS is reversible.

## Discussion

We demonstrate here that LTD induced by low-frequency stimulation of the somatic neuromuscular junction produces lasting refractoriness of synaptic vesicle recycling. Labeling with the fluorescent styryl dye FM2-10 revealed that readily releasable (RRP) and total recycling pool (TP) sizes were normal in LTD. However, loading of the RRP to saturation required a longer period of stimulation and FM2-10 destaining experiments indicated reduced synaptic vesicle exocytosis in LTD. Specifically, induction of LTD by low-frequency stimulation reduced exocytosis from the RRP, which then slowly recovered towards baseline levels following a period of non-stimulation (2 hours). We therefore provide direct evidence for refractory synaptic vesicle exocytosis following induction of a skeletal muscle-dependent form of LTD.

Transmitter release is the result of multiple convergent presynaptic processes. Presynaptic depression can result from reduced RRP size, reduced refilling of vesicle pools by endocytosis, reduced Ca^2+^ availability/responsiveness or modified activity of the exocytotic machinery (for review see [26]–[29]). Altered releasable pool size has been implicated in several forms of neuromuscular plasticity [23], [30]–[32], however our results argue against transfer of vesicles between the resting, recycling and readily releasable pools induced by this form of LTD. In our control muscles, the RRP constituted approximately 20% of the total recycling pool (Fig. 2*B*), consistent with previous work at the amphibian neuromuscular junction [18], [19]. The level of RRP labeling in LTD was the same as controls, whether expressed in absolute fluorescent units (Fig. 2*B*) or as a proportion of the total recycling pool (Fig. 2*C*). The size of the total recycling pool was also unchanged by LTD (Fig. 2*B*). By contrast, both loading and unloading of dye from the RRP took longer in LTD (Fig. 2*C*, 3*B*). Refractory unloading of dye from the RRP has also been demonstrated in NO-mediated hippocampal LTD [24], [33], and at the NMJ in response to exogenous NO [34]. We conclude that our form of LTD specifically targets the availability, but not the total size, of the readily releasable vesicle pool (RRP).

A reduction in endocytosis can potentially decrease the availability of RRP vesicles and on first investigation our FM2-10 loading experiments, which show refractory dye loading in LTD, are consistent with this possibility. However, refractory dye loading can be a function of either reduced vesicle exocytosis or reduced vesicle endocytosis (or a combination of the two). Although we cannot rule out modulation of endocytosis during LTD (e.g. vesicles in depressed terminals being preferentially endocytosed into cisternae that are unavailable for destaining during subsequent stimulation [19], [35]), our findings favor a change to the exocytotic process as the dominant presynaptic mechanism underlying LTD. We have previously demonstrated profound (>40%) LTD of EPPs evoked by single APs fired at very low frequency (0.2 Hz, [13]). At the neuromuscular junction, an average of less than one vesicle out of a pool of ∼270 vesicles is released per active zone per stimulus [36] and the vesicle recycle time is approximately 75 s [37]. Thus, at these low stimulation frequencies, endocytotic pathways would likely be functioning below maximum capacity and disruption to endocytosis seems unlikely to be the major mechanism underlying the large reduction in EPP amplitude observed.

How, then, might LTD alter synaptic vesicle exocytosis? Alteration to presynaptic Ca^2+^ dynamics, via modulation of voltage-sensitive channels or alterations to nerve terminal Ca^2+^ buffering, is a common target of neuromuscular plasticity [15], [31], [38], [39]. While we did not measure the presynaptic Ca^2+^ signal directly, our observation that paired pulse facilitation (PPF) is unchanged in LTD (Fig. 1*B*) is inconsistent with a significant change in presynaptic Ca^2+^ signaling during the depression. Short-term synaptic facilitation (enhancement of transmitter release in response to repetitive stimulation at short time intervals) is highly dependent on presynaptic Ca^2+^ handling [27], [40], thus normal PPF is unlikely in the face of even a small reduction in presynaptic Ca^2+^ influx.

Instead, we propose that the LTD acts to provide a break on the vesicle release machinery at a late stage of exocytosis, downstream of the presynaptic Ca^2+^ signaling that triggers exocytosis and underlies PPF. This finding provides important direction for future work exploring the presynaptic target(s) of neuromuscular LTD, as many of the most thoroughly explored pathways target voltage-gated Ca^2+^ or K^+^ channels that modulate transmission upstream of Ca^2+^ entry [41]. Of the mechanisms for presynaptic inhibition of transmitter release downstream of Ca^2+^ entry, we suggest that a signaling pathway implicated in LTD of glutamatergic transmission at hippocampal synapses warrants particular investigation. In this form of LTD (reviewed in [42]), activation of presynaptic G-protein coupled receptors, including mGluRs and A1 adenosine receptors (both established modulators of neuromuscular transmission [8], [43]), is proposed to promote binding of Gβγ to the C-terminus of the target-membrane soluble NSF attachment receptor (SNARE) protein SNAP25. Gβγ competes with the calcium-sensing vesicle SNARE synaptotagmin for binding with SNAP-25, thus inhibiting exocytosis [44], [45]. Of particular interest here, this LFS-dependent hippocampal LTD requires the concomitant retrograde action of NO, produced in response to activation of neuronal nitric oxide synthase, to activate presynaptic soluble guanylyl cyclase and generate cGMP [24]. NO signaling, as implicated in this form of neuromuscular LTD by our previous pharmacological experiments [13], appears necessary for converting short-term depression of hippocampal transmitter release into LTD [46]. Our pharmacological experiments indicated that maintenance of neuromuscular LTD requires sustained elevation of NO signaling, possibly via dephosphorylation of muscle neuronal NO synthase by calcineurin [13]. In the context of the above signaling pathway, this sustained NO signaling would be expected to maintain the cGMP elevation required for long-term refractoriness of exocytosis in LTD [46], [47]. SNAP-25 is already implicated in short-term depression at the NMJ [48] and it will be interesting to see if an additional role for SNAP-25 modulation in neuromuscular long-term depression will emerge, as recently observed in the hippocampus.

The somatic NMJ differs from most synapses because its primary functional requirement is reliability, rather than nuanced integration of subthreshold synaptic potentials. There is now substantial evidence for precise homeostatic control of synaptic transmission by a multiplicity of cell intrinsic and intercellular synaptic mechanisms (for review see [15], [49]–[53]), which are likely to contribute to this synaptic reliability. However, these precise homeostatic processes may act in parallel with feedback mechanisms that maintain synaptic processes within broad functional limits but do not fit the classical definition of homeostatic plasticity, where baseline synaptic transmission is restored (see, for example [54]). We speculate that the LTD provided here may fulfill such a role, constraining transmitter release at the NMJ to a functional, sustainable level. The involvement of NO-mediated plasticity in transmitter conservation at the amphibian NMJ, via a different cellular mechanism, has recently been postulated [10].

Our LTD is well suited to such a role, as it provides reversible depression of presynaptic release at all terminals innervating a muscle fiber in response to repetitive muscle AP firing [9]. Furthermore, the LTD acts at a late stage of transmitter release and therefore does not impede other forms of plasticity (e.g. PPF) that may be important for activity-dependent modulation of synaptic function (see discussion by [26], [31]). Indeed, presynaptic modulation downstream of calcium influx, as implicated in our LTD, is proposed as an important contributor to synaptic stability at the NMJ [55]. We have shown that PPF at high frequencies restores the reliability of neuromuscular transmission (Fig. 1*C*), so it seems that during *in vivo* patterns of synaptic activity (short, high-frequency bursts repeated at low-frequency [56]) the LTD could restrain functionally redundant exocytosis without a cost to synaptic reliability.
